# *CCM3/SERPINI1* bidirectional promoter variants in patients with cerebral cavernous malformations: a molecular and functional study

**DOI:** 10.1186/s12881-016-0332-0

**Published:** 2016-10-13

**Authors:** Concetta Scimone, Placido Bramanti, Alessia Ruggeri, Luigi Donato, Concetta Alafaci, Concetta Crisafulli, Massimo Mucciardi, Carmela Rinaldi, Antonina Sidoti, Rosalia D’Angelo

**Affiliations:** 1 Department of Biomedical and Dental Sciences and Morphofunctional Imaging, Division of Medical Biotechnologies and Preventive Medicine, University of Messina, via C. Valeria 1, 98125 Messina, Italy; 2 Department of Cutting-Edge Medicine and Therapies, Biomolecular Strategies And Neuroscience, Section of Neuroscience-applied, Molecular Genetics and Predictive Medicine, I.E.ME.S.T, Palermo, Italy; 3 IRCCS Centro Neurolesi “Bonino-Pulejo”, Messina, Italy; 4 Department of Economy, University of Messina, Messina, Italy

**Keywords:** Bidirectional promoter, *CCM3/SERPINI1*, Polymorphism, Association study, Dual luciferase-assay, CCM genes

## Abstract

**Background:**

Cerebral cavernous malformations (CCMs) are vascular anomalies of the nervous system mostly located in the brain presenting sporadically or familial.

Causes of familial forms are mutations in *CCM1 (Krit1), CCM2 (MGC4607)* and *CCM3 (PDCD10)* genes. Sporadic forms with no affected relative most often have only one lesion and no germ line mutations. However, a number of sporadic cases with multiple lesions have been reported and are indeed genetic cases with a de novo mutation or a mutation inherited from an asymptomatic parent.

**Methods:**

Here, we performed an analysis of regulatory region of CCM genes in 60 sporadic patients, negative for mutations in coding region and intron-exon boundaries and large deletion/duplications in CCM genes by direct sequencing and MLPA. Among 5 variants identified in 851-bp region shared by *CCM3* and *SERPINI1* genes and acting as asymmetric bidirectional promoter, two polymorphisms c.-639 T > C/rs9853967 and c.-591 T > C/rs11714980 were selected. A case-control study was performed to analyze their possible relationships with sporadic CCMs. Promoter haplotypes activities on *CCM3/SERPINI1* genes expression were tested by dual-luciferase assay.

**Results:**

No variants were identified in *CCM1* and *CCM2* regulatory regions. In *CCM3*/*SERPINI1* asymmetric bidirectional promoter 5 variants, 2 of them unknown and 3 corresponding to polymorphisms c.-639 T > C/rs9853967, c.-591 T > C/rs11714980 and c.-359G > A/rs9834676 were detected. While rs9853967 and rs11714980 polymorphisms fall in a critical regulatory fragment outside the minimal promoter in intergenic region, other variants had no effects on transcription factor binding according to RegRNA tool. Case-control study performed on 60 patients and 350 healthy controls showed frequencies of the mutated alleles significantly higher in the control group than in patients. Furthermore, the functional assay showed a significant reduction of *CCM3* expression for C-C haplotype even more than for T-C and C-T haplotypes. In *SERPINI1* direction, the reduction was not statistically significant.

**Conclusions:**

Our data indicated that rs9853967 and rs11714980 polymorphisms could be associated with a protective role in CCM disease.

## Background

Cerebral cavernous malformations (CCMs) are among the most common cerebral vascular anomalies characterized by clusters of enlarged blood vessels consisting of a single layer of endothelium and lacking smooth muscle and elastic tissue, without intervening brain parenchyma [1, 2]. CCMs, reported in up to 0.5 % of the population, are primarily found within the central nervous system where they result in increased risk for stroke, seizures, recurrent headaches and focal neurological deficits [1–4]. CCMs are associated with loss-of-function mutations in any one of the three CCM genes CCM1/KRIT1, CCM2/MGC4607 or CCM3/PDCD10 and occur in both sporadic and familial forms [5], inherited in an autosomal dominant fashion with a high penetrance.

Familial CCM accounts for only 20 % of cases but tends to be more severe than sporadic CCM [1], with patients exhibiting multiple lesions and increased hemorrhage rates.

More than 150 distinct *CCM1*, *CCM2*, and *CCM3* germline mutations are known [6–9].

Linkage analyses predicts that 40 % of patients with familial forms is linked to CCM1 locus, 40 % to CCM3 and, only 20 % to CCM2 [10–13]; however, according with reported experimental data, these frequencies are not confirmed by an our recent screening of a cohort of Italian patients with CCMs [9]. Our data showed that 54 % of patients leads CCM1 mutations, a very lower percent was observed for CCM3 (6 %) while 18 % leads mutation at CCM2 locus; the absence of any mutation in 22 % of patients, leads to consider other possible elements involving in the development of disease like a somatic mosaicism of a de novo mutation that occurred during gestation and is not detectable in DNA extracted from peripheral blood [5, 6, 14], the possibility of large deletions or duplications not detected by direct sequencing or mutations located in regulatory regions of CCM genes, and finally, the existence of other as yet unidentified genes.

No data in the literature exist on CCM genes regulatory regions. Therefore we focused our attention on molecular analysis of these regions in a cohort of CCM sporadic patients.

Initially we examined the promoters of *CCM1* and *CCM2* genes, located about 4.0 kb (*CCM1*) and 0.5 Kb (*CCM2*) upstream from the ATG start codon, and finally *CCM3* one. This is an evolutionary conserved bidirectional promoter shared with *SERPINI1* gene, coding for a serine protease inhibitor [15]. Two genes were found to be closely adjacent to each other in a head-to-head orientation and separated by an exceptionally short intergenic region of 851-bp that function as a bidirectional promoter to regulate transcription of both genes. A 175-bp fragment from nt1 to 175 in the vicinity of *CCM3* was further determined to function as a minimal bidirectional promoter. A critical regulatory fragment, from nt 176–473 outside the minimal promoter in the intergenic region, was identified to contain a strong repressive element for *SERPINI1* and an enhancer for CCM3 [15]. In a parallel study, through an in-silico approach, we investigated the existence of common pathways to both genes in order to explain the need of this co-regulation and to detect a possible involvement of *SERPINI1* in CCM pathogenesis (data not shown).

Here, we focused on intergenic region of 851 bp shared by *CCM3* and *SERPINI1*, where we identified 5 variants, 2 of them unknown (c.-964 G > C and c.-419G > T) and 3 corresponding to polymorphisms: c.-639 T > C/rs9853967, c. -591 T > C/rs11714980 and c.-359G > A/rs9834676.

RegRNA analysis showed that the rs9834676 polymorphism and two novel variants do not affect the transcriptional regulatory motifs. Furthermore, literature data report rs9853967 and rs11714980 polymorphisms falling in a critical regulatory fragment outside the minimal promoter in intergenic region [15].

Therefore, our attention has focused on rs9853967 and rs11714980 polymorphisms and their possible association with CCM disease. A case-control study was carried-out and the polymorphisms effects on two genes expression were determined by a functional assay.

## Methods

### Subjects

A total of 60 CCM patients (40 % men, 60 % women) were recruited during 2003–2013.

Detailed clinical and neuroimaging information on patients and their relatives were collected through direct interview by review of the medical charts, before CCM gene molecular analysis and after providing their written informed consent. On the basis of pedigree analysis and in the absence of relatives positive to biomolecular investigation and/or Magnetic Resonance Imaging (MRI) (standard spin echo and fast turbo spin echo T1- and T2-weighted axial, coronal, and/or sagittal images) the patients were considered sporadic. Lesions were single in 58 patients (97.0 %) and multiple in 2 patients (3.0 %).

The age of symptoms onset, in all patients examined, coincides with the age at diagnosis and the patient*'*s age at the time of recruitment. The mean age at onset of symptoms was 39,5 ± 18,7 years (median, 36.0 years; range, 10–73 years) (Table 1). A group consisted of 350 unrelated, randomly selected, ethnically matched, healthy individuals were recruited. Demographic data on controls are summarized in Table 1. Age at symptoms onset of male vs female was matched in the CCM patients, as well as age of male versus female in the controls. Control individuals were selected to match the CCM patients for age. Table 1 indicates no significant differences (*p* > 0.05). This study was approved by the Scientific Ethics Committee of the Azienda Ospedaliera Universitaria*–*Policlinico ‘G. Martino’ Messina. Informed consent was obtained from all patients and controls.

**Table 1:** Demographic and disease data on CCM sporadic patients and controls

(A) CCM sporadic patients (*n* = 60)					
		Age of symptoms onset			
Gender	*n*	Mean ± SD	Range		Lesion number
Male	24	35,6 ± 19,1	10–71		2/60 > 1 lesion
Female	36	42,2 ± 18,3	15–73		
Overall	60	39,5 ± 18,7	10–73		
*Male + Female (asymptomatic)*	2 + 2				
(B) Healthy controls (*n* = 350)					
			Age		
	*n*	Mean ± SD	Range		
Male	204	44,3 ± 17,9	12–73		
Female	146	42,3 ± 18,3	12–73		
Overall	350	43,5 ± 18,1	12–73		

*p* > 0.05 indicates no significant differences. Statistical analysis was tested by the Student’s *t*-test. *CCM*, cerebral cavernous malformation; *SD*, standard deviation

The age of symptoms onset in all patients (with the exception of asymptomatic patients) coincides with the age at diagnosis

### Genetic analysis of the CCM1, CCM2 and CCM3 promoters

DNA was extracted from leukyocytes by using the standard protocols. The amplification of regulatory regions of *CCM1* and *CCM2* genes was performed using the primers pairs designed according to the published nucleotide sequence of GenBank (accession no. NG_012964.1 *CCM1*; NG_016295.1 *CCM2*) (Table 2).

**Table 2:** Primers for PCR amplification of CCM genes promoter regions

	Primer set	Anneal (°C)
^a^ *CCM1*	F: 5’ – ACA GAC AGA ACA ACA ATG CTC – 3’ R: 5’ – TCA CTG GAC CTG CAG TCT CT – 3’	58
^a^ *CCM2*	F: 5’ – TTT ACA TTC TAG CTG TGC TA – 3’ R: 5’ - CTG GAC AGG TGC GTT CTC – 3’	49
*CCM3- SERPINI1*	F: 5’ - TGAGGCACTGACTTCACTT-3’ R: 5’-CTTAGCTGCTCTCAGGGA-3’ (P1)	58
	F: 5’ - TCCCTGAGAGCAGCTAAG-3’ R: 5’ - GCTCTCGTTCCTGCTTTC-3’ (P2)	56

^a^Primers were designed to amplify a promoter region located about 3.8 kb (*CCM1*) and 0.5 kb (*CCM2*) from the ATG start codon

Polymerase chain reaction (PCR) was carried out in a thermal cycler (Gene Amp PCR System 2700; PE Applied Biosystems, Foster City, CA) under following conditions: 0.8 μg of genomic DNA was added to 50 μl reaction mixture containing a 0.2 μm concentration of each primer and 1 U Taq Gold polymerase (PE Applied Biosystems).

PCR conditions for *CCM1* promoter region (655 bp) were as follows: denaturation at 95 °C for 45 s, annealing at 58 °C for 35 s and extension at 72 °C for 40 s for 35 cycles, after an initial 10 min denaturation at 95 °C.

For *CCM2* promoter region (620 bp) we employed these conditions: one cycle of denaturation at 95 °C for 10 min, followed by 35 cycles at 94 °C for 40 s, 49 °C for 30 s, and 72 °C for 45 s, before a final extension at 72 °C for 10 min.

Short intergenic region (851 bp) between two non-homologous genes, *SERPINI1* and *CCM3*, was amplified using two primer sets designed according to published sequence data (Accession no. NG_ 008217.1 for *SERPINI1*; NG_008158.1 for *CCM3*) and shown in Table 2.

Two fragments of 580 bp (P1) and 383 bp (P2) were amplified under following conditions: for P1 denaturation at 94 °C for 50 s, annealing at 58 °C for 45 s and extension at 72 °C for 50 s for 35 cycles, after an initial 10 min denaturation at 95 °C; for P2 denaturation at 94 °C for 40 s, annealing at 56 °C for 35 s and extension at 72 °C for 45 s for 35 cycles, after an initial 10 min denaturation at 95 °C.

PCR products were sequenced by direct sequencing. The nucleotide number relative to polymorphisms identified in intergenic region of 851 bp was indicated respect to the transcriptional start sites of the reference sequence reported by the NCBI database (in direction of *SERPINI1*).

### Statistical analysis

Analysis of data was performed using computer software Statistical Package for Social Science (SPSS) for Windows (Version 6.0.1) and Epi Info (version 6.0.4).

Comparisons between means of age of symptoms onset of male versus female in the CCM group and overall CCM age at onset versus overall healthy control age were calculated with the Student’s *t*-test. For each group (control and patients), allele frequencies were calculated by direct gene counting. Estimates of statistical significance were calculated by standard *χ* ^2^ analysis for one degree of freedom. Descriptive analysis included Student’s *t*-test of means and the respective standard deviation (SD) for cases and controls. A two-sided probability value of < 0.05 was considered to indicate statistical significance. The results from dual-luciferase assay were compared using one-way ANOVA for repeated measurements.

### In silico analysis

RegRNA (http://regrna.mbc.nctu.edu.tw/html/prediction.html) [16], a transcription factor prediction tool, was used to to evaluate how the identified variants could interfere with the transcriptional regulatory motifs in *CCM3/SERPINI1* promoter.

The relative position numbers of the nucleotides in this intergenic region of 851 bp was as follows: 9 (- c.964G > C), 335 (-c.-639 T > C), 383 (-c.-591 T > C), 556 (- c.-419 G > T), 615 (-c.-359G > A).

### Functional studies

#### Cell culture

U373-MG (human, Caucasian, glioblastoma-astrocytoma) cells were cultured in Dulbecco’s modified Eagle’s medium (DMEM) supplemented with 10 % Fetal Bovine Serum (FBS), 100U/ml of penicillin and 1 mg/ml of streptomycin (Lonza) at 37 °C in a water-satured atmosphere with 5 % CO_2_.

### Construction of the reporter gene plasmids

To examine the potential effects of the c.-639 T > C and c.-591 T > C on the intergenic region of 851 bp transcription activity, we compared the promoter activity of four common haplotypes (c.-639 T/c.-591 T) (T-T), (c.-639C/c.-591 T) (C-T), (c.-639 T/c.-591C) (T-C), (c.-639C/c.-591C) (C-C). 851 bp intergenic regions were amplified by PCR using genomic DNA from donors carrying each haplotype, using the primers showed in the Table 3 and under the following conditions: 1 cycle of 95 °C for 5 min; 35 cycles of 94 °C for 40 s, 53 °C for 35 s, and 72 °C for 40 s; and 1 cycle of 72 °C for 10 min.

**Table 3:** Primer sequences used to amplify *CCM3* promoter region for subsequent cloning

	Primer sequence	
*SERPINI1* (1-851)	F: 5’ATAGATCTACTCCGGCGACGCCGGA-3'	R: 5’ATAAGCTTGTCCAGACTGCGCCTCT-3’
*CCM3* (851-1)	F: 5’-ATAGATCTGTCCAGACTGCGCCTCT-3’	R: 5’-ATAAGCTTACTCCGGCGACGCCGGA-3’

PCR products were inserted upstream of the luciferase gene cloned into the pGL4.10 (luc2).

For this purpose, PCR products, as well as pGL4.10 (*luc2*) were digested with *BglII* and *HindIII* (Promega) and then purified (PureLink™ PCR Purification Kit, Life Technologies). Vector arms were ligated overnight to the digested PCR fragment. The novel constructs were subcloned into E. coli Top 10 cells (Life Technologies) and single colonies were miniprep. The correct sequence of all the clones was verified by DNA sequencing.

### Transient transfection and promoter assays

Cells were first seeded in 96-well culture plates at a density of 2 × 10^4^ cells per well. In each well, cells were then co-transfected with 0.6 μl of FuGENE HD Transfection Reagent (Promega) and a mixture consisting of 0.2 μg of the pGL4.10 [*luc2*] promoter constructs and 0.02 μg of the co-reporter vector pGL4.74 [*hRluc/*TK] in a serum-free medium and then incubated for 24 h at 37 °C in a humidified atmosphere of 5 % CO_2_ in air. After incubation, cells were washed twice with PBS and lysed by Passive Lysis Buffer (Promega). Luciferase activity was measured using Dual Luciferase assay kit (Promega) and GloMax-Luminometer (Promega). Reporter construct activity was normalized by comparison with activity from the *Renilla* luciferase construct. Luciferase activities are representative of at least three independent experiments, with each construct tested in triplicate per experiment.

## Results

### Genotype and allele frequencies of c.-639 T > C/ rs9853967 and c.-591 T > C/ rs11714980 polymorphisms in patients and controls

In the promoter regions of *CCM1* and *CCM2* of 60 CCM patients cohort, no variants has been identified.

Conversely, analysis of regulatory intergenic region shared by *CCM3* and *SERPINI1* has lead to the identification of 5 variants, 2 unknown (c.-964 G > C and c.-419G > T) and 3 reported such as polymorphisms in SNP database (http://www.ensembl.org/Homo_sapiens/Gene/Sequence?db=core;g=ENSG00000163536;.r=3:167453031-167543356: c.-639 T > C/rs9853967, c.-591 T > C/rs11714980 and c.-359 G > A/rs9834676).

To evaluate the possible influence that these variants could have on transcriptional regulatory motifs, wild-type (c.- 964G, c.-639 T, c.-591 T- c.-419 G, c.-359G) and mutated (c.- 964C, c.-639C, c.-591C, c.-419 T, c.-359A) sequences of intergenic region shared by *CCM3* and *SERPINI1* were analyzed by using RegRNA. In mutated sequence the loss of transcriptional regulatory motif located in position 334 ~ 338 (RegRNA ID:R0146) was detected (http://regrna.mbc.nctu.edu.tw/php/showtable.php?ColorRegion=&FileDir=tmp/20160620/200730&SeqID=200730&MotifInfo=Transcriptional%20Regulatory%20Motifs&MotifType=tfbs) respect to wild-type one (http://regrna.mbc.nctu.edu.tw/php/showtable.php?ColorRegion=&FileDir=tmp/20160620/200521&SeqID=200521&MotifInfo=Transcriptional%20Regulatory%20Motifs&MotifType=tfbs). To exclude a possible role of variants c.-964 G > C, c.-419G > T and c.-359G > A in the loss of this motif a further analysis was performed: the motif was absent also in (c.- 964G, c.-639C, c.-591C, c.-419G,-c.-359G) sequence, assigning to c.-639 T > C and c.-591 T > C polymorphisms a role in the loss (http://regrna.mbc.nctu.edu.tw/php/showtable.php?ColorRegion=&FileDir=tmp/20160620/204528&SeqID=204528&MotifInfo=Transcriptional%20Regulatory%20Motifs&MotifType=tfbs). Furthermore, literature data report rs9853967 and rs11714980 polymorphisms falling outside the minimal promoter (from nt 176–473) in a critical regulatory element identified to contain a strong repressive element for *SERPINI1* and an enhancer for *CCM3* [15].

Because no data about these polymorphisms and CCM there are in literature and few data about polymorphisms association and CCM too [17], we focused our attention on above mentioned rs9853967 and rs11714980.

First, we analyzed c.-639 T > C/ rs9853967, c.-591 T > C/rs11714980 polymorphisms frequencies in a group of CCM sporadic patients vs a healthy control sample. Genotype distribution is given in Table 4. About the -639 T > C/rs9853967 polymorphism, 35/350 (10 %) controls typed were homozygous for the mutant ‘C’ allele. None was found to be homozygous in the patient group; 6 heterozygotes (10 %) were found among 60 CCM patients vs the 139/350 (40 %) in control group.

**Table 4:** Allele and genotype frequencies of *CCM3-SERPINI1* promoter polymorphisms

	CCM patients		Control		*χ*2	*p*-value
	No.	(%)	No.	(%)		
c.- 639 T > C						
TT	54	(90)	176	(50)		
TC	6	(10)	139	(40)	33.2	0.0001
CC	0		35	(10)		
T	114	(95)	491	(70)	32.7	0.0001
C	6	(5)	209	(30)		
c.-591 T > C						
TT	53	(88)	176	(50)	30.6	0.0001
TC	7	(12)	138	(39)		
CC	0		36	(10)		
T	113	(94)	490	(70)	30.7	0.0001
C	7	(6)	210	(30)		

Position relative to the first position of the starting codon ATG (in direction of *SERPINI1*)

The frequency of the ‘C’ allele was 0.3 for controls and 0.05 for patients (*χ* ^2^ = 32.7; p = 0.0001).

Same trend was observed for c.-591 T > C/ rs11714980 polymorphism with “C” mutated allele having a frequency in CCM samples of 0.06 %.

The second step regarded the characterization of the effects of the c.-639 T > C/ rs9853967 and c.-591 T > C/rs11714980 polymorphisms.

For this purpose two constructs of 851 bp were made: toward the *CCM3* and the *SERPINI1* direction.

**Fig. 1:**
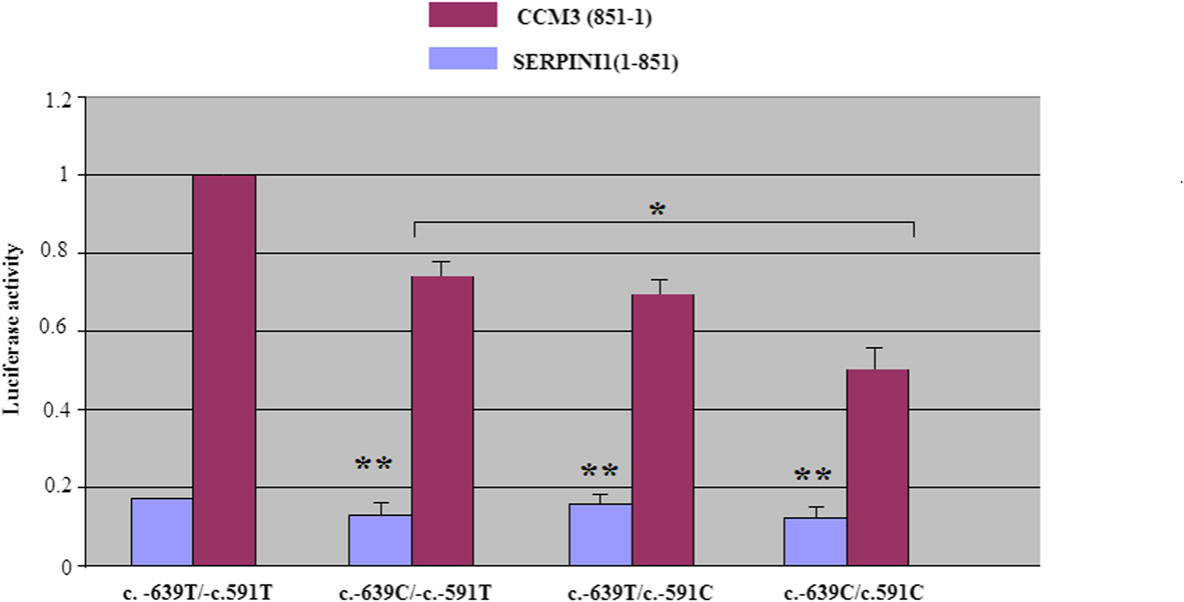
Transcription activity analysis of *CCM3/SERPINI1* haplotypes of the c.-639 T > C and c.-591 T > C polymorphisms. The transcription activity was measured using the Dual-Luciferase Reporter Assay System in U373 MG (human, Caucasian, glioblastoma-astrocytoma) cells. Luciferase activities are representative of at least three independent experiments, with each construct tested in triplicate per experiment. *p < 0.01; **p > 0.05

Promoter activity of the four common haplotypes (T-T, T-C, C-T and C-C) was compared. The T-C and C-T haplotypes significantly decreased promoter activity compared with the T-T haplotype; C-C haplotype showed largest reduction of promoter activity (*p* < 0.01). In both cases this reduction was only in *CCM3* direction. In *SERPINI1* direction, it was not statistically significant (*p* > 0.05) (Fig. 1).

## Discussion

We performed a molecular analysis on CCM genes regulatory regions in a cohort of CCM sporadic patients negative for mutations in the coding regions of these genes as well as for large genomic rearrangements not detectable to direct sequencing.

Case-control study performed on 60 patients and 350 healthy controls showed a possible association with CCM disease. Frequencies of the mutated alleles were significantly higher in the control group than in patients suggesting a possible protective effect of two polymorphisms.

Dual-luciferase assay of the c.-639 T > C/rs9853967 and c.-591 T > C/rs11714980 polymorphisms falling in the critical regulatory fragment of bidirectional promoter *SERPINI1* and *CCM3*, showed a significant reduction of *CCM3* expression for C-C haplotype even more than for T-C and C-T haplotypes. In *SERPINI1* direction, the reduction was not statistically significant.

*CCM3* reduced expression would seem to disagree with case-control study data showing a higher frequency of the mutated alleles in control group than in patients. This taking into account the only apoptotic role of CCM3 protein and the association between *CCM3* loss-of-function mutations and CCM development [18, 19].

Evidences suggest that CCM3 interacts with CCM1 and CCM2 proteins in an intracellular complex sharing some common signaling pathways [20–23].

Furthermore, it is involved in angiogenesis and remodeling of cerebral vessels. Thus CCM3 protein acts as a positive regulator of Ste20-related kinase MST4 [24] promoting cell growth and transformation via extracellular signal-regulated kinase (ERK) pathway.

These processes are the basis of pathogenesis, progression, and oncogenic behavior of human cancers [25] as well as of CCM pathogenesis. Furthermore, it was shown that potent short interfering RNAs (siRNAs) against *CCM3* and MST4 are able to specifically inhibit the expression of *CCM3* and MST4 mRNA, respectively [26–28].

Therefore, based on these observations it is possible to assume that c.-639 T > C/rs9853967 and c.-591 T > C polymorphisms in regulatory intergenic region shared by *CCM3* and *SERPINI1* genes could have a protective role against CCM pathogenesis. In fact, two polymorphisms cause a considerable reduction of *CCM3* expression to dual luciferase-assay, thus justifying the higher frequencies of mutated alleles found in the control group than in patients. The reduced expression of *CCM3* gene we observed might be accompanied by reduced ERK activity and attenuated cell growth. Further studies are needed to confirm this.

## Conclusions

In the present study, for the first time, we found that c.-639 T > C/rs9853967 and c.-591 T > C/rs11714980 polymorphisms in *CCM3*/*SERPINI1* genes asymmetric bidirectional promoter were able to determine a sensible reduction of *CCM3* expression. Mutated allelic frequencies higher in control group than in patients and the reduction of *CCM3* expression especially in C-C haplotype carriers suggesting a possible protective role of these polymorphisms in CCM pathogenesis.

## Abbreviations

CCMs: Cerebral cavernous malformations
DMEM: Dulbecco’s modified Eagle’s medium
ERK: Extracellular signal-regulated kinase
FBS: Fetal Bovine Serum
MRI: Magnetic resonance imaging
PCR: Polymerase chain reaction
SD: Standard deviation
siRNAs: Short Interfering RNAs
SPSS: Statistical package for social science
